# Monitoring luciferase-labeled human prostate stem cell antigen-expressing tumor growth in a mouse model

**DOI:** 10.3892/etm.2013.1293

**Published:** 2013-09-13

**Authors:** LEI DONG, XIAOPENG ZHANG, CHANGMING YU, TING YU, SHULING LIU, LIHUA HOU, LING FU, SHAOQIONG YI, WEI CHEN

**Affiliations:** 1 Clinical Laboratory Center, PLA Air Force General Hospital, Haidian, Beijing 100142;; 2 Beijing Institute of Biotechnology, Academy of Military Medical Sciences, Fengtai, Beijing 100071, P.R. China

**Keywords:** bioluminescence, luciferase, prostate cancer, prostate stem cell antigen, tumor model

## Abstract

The aim of this study was to establish a tumor model in mice with the expression of luciferase (Luc) and human prostate stem cell antigen (PSCA), in order to evaluate the activities of anticancer drugs or vaccines for prostate cancer. RM-1 cells were stably transfected with pcDNA-Luc and pcDNA-PSCA plasmids. The Luc-expressing cells were examined using a luminometer and the PSCA-expressing cells were examined using a reverse transcription-polymerase chain reaction (RT-PCR) and flow cytometric analysis. Male C57BL/6 mice were inoculated subcutaneously with the RM-PSCA/Luc cells, prior to the tumor growth and survival time of the mice being measured, respectively. *In vivo* bioluminescence imaging was used to detect Luc expression and immunohistochemical analysis was used to detect PSCA expression. Inoculation of the tumor cells into the C57BL/6 mice closely mimicked the tumor growth of prostate cancer. All of the inoculated mice exhibited a detectable tumor within two weeks. Tumor progression was able to be quantitatively monitored following the inoculation of 1×10^6^ RM-PSCA/Luc cells. There was an excellent correlation (R^2^=0.9849) between the photon counts and tumor volume. The expression of PSCA in tumor tissues was confirmed using immunohistochemical analysis. The Luc and PSCA co-expression tumor model was successfully established in mice, which is likely to accelerate the understanding of the pathogenesis of prostate cancer and facilitate the development of novel antitumor drugs or vaccines for the disease.

## Introduction

Prostate cancer is the most common types of noncutaneous cancer with a high mortality rate in American males (1). Despite the significant progress that has been made in the treatment of the disease, therapeutic options for advanced and metastatic prostate cancer remain unsatisfactory (2,3). The absence of effective therapies for prostate cancer has entailed an intensive search for novel anticancer strategies. In recent years, rapid progress has increased the understanding of prostate cancer immunotherapy.

Prostate stem cell antigen (PSCA), first described by Reiter *et al* (4), is a surface glycoprotein that is upregulated in androgen-dependent and -independent prostate cancer xenografts and downregulated in the normal prostate (4). The PSCA gene encodes a 123-amino acid protein that is a glycosylphosphatidylinositol (GPI)-anchored cell surface antigen associated with the Thy-1/Ly-6 family. Binding to cellular membranes with covalent linkages, which may be degraded by phosphatase (5,6). In addition, there is a direct correlation between the expression level of PSCA and the tumor stage and grade and the bone metastases (7). To date, numerous studies have indicated that a vaccination based on PSCA enhances the cytotoxic T lymphocyte (CTL) response and inhibits PSCA^+^ tumor growth in mice (8–10).

Animal models are important tools to facilitate an enhanced understanding of cancer biology and may be used to evaluate the activities of investigational agents. Xenografts of human tumor cell lines inoculated subcutaneously into mice have been used to investigate cancer treatment since the late 1950s (11). However, traditional animal models typically require the sacrifice of the animal. Furthermore, it is not possible to visualize the growth and metastasis of the tumor and there is a lack of sensitivity (12). Therefore, novel sensitive methods of detecting and monitoring *in vivo* tumor growth and metastatic disease are required, with less invasive approaches.

Whole-body fluorescence and bioluminescence imaging have transformed the study of gene expression and protein function by enabling external visualization using sensitive detection systems (13–15). Cancer cell lines stably transfected either with the firefly luciferase (Luc) or green fluorescent protein have been used to monitor local tumor growth and metastasis in living mice (16).

In the present study, we have investigated for the first time, to the best of our knowledge, the feasibility of whole-body bioluminescent reporter imaging for the visualization of the *in vivo* development of local tumor growth following the inoculation of Luc and PSCA co-transfected RM-1 cells (RM-PSCA/Luc), a prostate cancer cell line. The results showed that it was possible to monitor tumor growth with noninvasive, sensitive and quantitative localization *in vivo* using whole-body bioluminescent reporter imaging.

## Material and methods

### Mice and cell lines

Male C57BL/6 mice (4–6 weeks old) were purchased from the Center for Laboratory Animals (Beijing, China). The mouse prostate tumor cell line RM-1, syngeneic to C57BL/6, was purchased from the Shanghai Cell Institute (Shanghai, China). This study was carried out in strict accordance with the recommendations in the Guide for the Care and Use of Laboratory Animals of the Academy of Military Medical Sciences (Beijing, China). The protocol was approved by the Committee on the Ethics of Animal Experiments of the Academy of Military Medical Sciences.

### Plasmid DNA constructs

All constructs were cloned into the pcDNA3.1(+) vector (Invitrogen Life Technologies, Carlsbad, CA, USA). The human PSCA gene was amplified from the vector pMD-PSCA with the following primers: 5′-CCC AAG CTT ACC ATG AAG GCT GTG CTG CTT-3′ and 5′-CCC GGA TCC CTA TAG CTG GCC GGG TCC-3′, and cloned into the *Hin*dIII and *Bam*HI sites of pcDNA3.1 to generate pcDNA-PSCA. To generate pcDNA-Luc, the Luc gene was amplified from the vector pGL3 with the following primers: 5′-CCG GCT AGC ATG GAA GAC GCC AAA AAC-3′ and 5′-CCG AAG CTT TTA CAC GGC GAT CTT TCC-3′, prior to being cloned into the *Hin*dIII and *Nhe*I sites of pcDNA3.1. PSCA amplification was performed for 3 min at 94°C, immediately followed by 30 sec at 94°C, 30 sec at 55°C and 30 sec at 72°C for 30 cycles. The reaction mixture of Luc was incubated for 3 min at 94°C, followed by 30 sec at 94°C, 30 sec at 55°C and 90 sec at 72°C for 30 cycles. An additional extension step was performed for 10 min at 72°C for Luc and PSCA, respectively. DNA sequencing was performed to confirm that all constructs had the desired sequence and open reading frame. Following this, pcDNA-PSCA or pcDNA-Luc was transformed into DH5α-competent *Escherichia coli*. Plasmid DNA copies were amplified in liquid culture and purified using a Plasmid Mini kit (Promega, Madison, WI, USA).

### Construction of stable transfectants expressing the Luc and PSCA reporter genes

To generate a Luc and human PSCA-expressing cell population, RM-PSCA/Luc, RM-1 was transfected with pcDNA-PSCA and pcDNA-Luc plasmids, followed by a Geneticin^®^ (G418) selection (Invitrogen Life Technologies). Subsequently, luminometry, reverse transcription-polymerase chain reaction (RT-PCR) and flow cytometry were used to detect the validity of these constructs. The expression of Luc was detected by luminometry. Following selection using Geneticin (G418), tumor cells were treated with cell culture lysis buffer. Having been mixed with luciferin at a ratio of 1:5, the tumor cells were then assessed for Luc expression. Tumor cells with luciferase activity >1 were reserved for the analysis of PSCA expression. For RT-PCR analysis, the following primers: 5′-TAA TAC GAC TCA CTA T-3′ and 5′-CTT GCC CAC GTA GTA G-3′ were used to amplify PSCA, while 5′-ACC ACA GTC CAT GCC ATC AC-3′ and 5′-TCC ACC ACC CTG TTG CTG TA-3′ were used for β-actin. Expression of PSCA on the cell surface was detected by staining the cell with anti-PSCA antibody (Santa Cruz Biotechnology, Inc., Santa Cruz, CA USA) and fluorescein isothiocyanate (FITC)-conjugated goat anti-rabbit immunoglobulin (Ig) G antibody (Santa Cruz Biotechnology, Inc.), followed by flow cytometric analysis.

### Murine model of human prostate cancer

Five 4 to 6-week-old male C57BL/6 mice were inoculated subcutaneously at the right flank with 1×10^6^ RM-PSCA/Luc cells. According to the result of our preliminary experiment, the expression of Luc was detectable using the luminometer when the RM-PSCA/Luc tumor cell population was 1×10^6^. Mice were imaged using whole-body bioluminescent reporter imaging for the first time one week subsequent to the inoculation of the cells, and this was followed by weekly imaging.

### Bioluminescent reporter imaging

The tumor growth was monitored using an imaging unit (IVIS Imaging System 50; Xenogen Corp., Alameda, CA, USA). The mice were anesthetized via an intraperitoneal injection of ketamine hydrochloride (0.66 mg/kg body weight) and xylazine (0.13 mg/kg body weight) in phosphate-buffered saline (PBS). Following this, an aqueous solution of luciferin was injected intraperitoneally. The mice were then placed in a light-obstructing chamber and the Luc-expressing cells were detected. The bioluminescent signal was quantified by measuring the number of highlighted pixels in the area shaped around each site of photon emission, with the aid of the imaging unit software.

### Tumor volume and survival time of mice

Following inoculation with RM-PSCA/Luc cells, the mice were monitored twice a week when the tumor was palpable. The tumor size was measured using vernier calipers and the tumor volume (V) was calculated according to the formula: V = 0.5a × b^2^, where a and b are the long and short diameters of the tumor, respectively. In addition, the survival time of the mice was recorded.

### Immunohistochemical examination

Tumor tissues were fixed overnight in 4% paraformaldehyde and the tissues were then transferred to 1:1 formaldehyde/ethanol for 1 h prior to being transferred to 70% ethanol until processing. Tissues were dehydrated using a graded ethanol series and embedded in paraffin wax at 58–60°C. The frozen tissue sections (4–6 *μ*m) were then washed for 10 min in dimethylbenzene twice for deparaffinization, 30 sec in 99% ethanol, 30 sec in 95% ethanol and 5 min in PBS. Following this, the sections were maintained at room temperature for 10 min with the addition of H_2_O_2_ and endogenous peroxidase activity was quenched. The tissue sections were then incubated for 24 h at 4°C with anti-PSCA polyclonal antibody (Santa Cruz Biotechnology, Inc.). Subsequent to being washed three times in PBS for 5 min, respectively, the sections were incubated for 20 min at 37°C with FITC-conjugated goat anti-rabbit IgG antibody (Santa Cruz Biotechnology, Inc.). This was followed by coloration with 3,3′-diaminobenzidine (DAB) using a DAB kit (Zhongshan Biotech Co., Beijing, China), in accordance with the provided instructions.

## Results

### Generation of the Luc and PSCA genes

The Luc and PSCA genes were amplified using PCR from the vectors pGL3 and pMD-PSCA, respectively. The sequencing of the Luc and PSCA genes was in accordance with the previous publications in GenBank (Luc cDNA, GenBank original accession no. AM295157; PSCA cDNA, GenBank original accession no. AF043498; http://www.ncbi.nlm.nih.gov/genbank/).

### Detection of stable transfectants expressing Luc and PSCA

**Figure 1.**
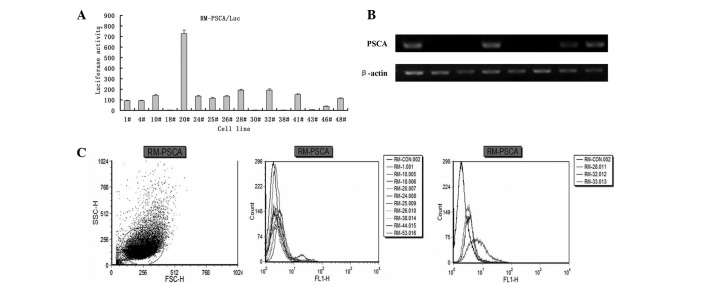
(A) Luminometry was used to test the expression of luciferase (Luc) *in vitro*. The y axis represents Luc activity. (B) Reverse transcription-polymerase chain reaction (RT-PCR) was used to test the expression of prostate stem cell antigen (PSCA) *in vitro*. RM-1 cells were transfected with pcDNA-PSCA and pcDNA-Luc plasmids via liposome. (C) Flow cytometric analysis of RM-1 cells and PSCA-Luc-cotransfected RM-1 cells using anti-PSCA polyclonal antibody. In order to detect surface PSCA expression only, cells were not permeabilized. The y axis represents cell number and the x axis represents fluorescent staining intensity on a logarithmic scale.

Following treatment with cell culture lysis buffer, RM-PSCA/Luc tumor cells were mixed with luciferin and then detected using a luminometer (Fig. 1A). The tumor cells with luciferase activity >1 subsequently underwent the test for PSCA expression using RT-PCR (Fig. 1B), prior to the expression of PSCA on the cell surface being detected using flow cytometry (Fig. 1C).

### Detection of the luciferase activity of RM-PSCA/Luc tumor cells

**Figure 2.**
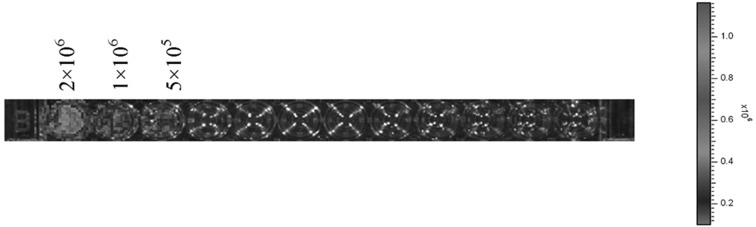
Detection of luciferase (Luc) activity of RM-PSCA/Luc tumor cells. The photon emission of Luc was easily detected by luminometry when the RM-PSCA/Luc tumor cell population was 1×10^6^. PSCA, prostate stem cell antigen.

RM PSCA/Luc tumor cells were prepared with serial 2-fold dilution from 2×10^6^ to 5×10^5^. The photon emission of Luc was detectable using a luminometer when the cell population of RM-PSCA/Luc was 1×10^6^ (Fig. 2).

### In vivo bioluminescence imaging of the prostate tumor

**Figure 3.**
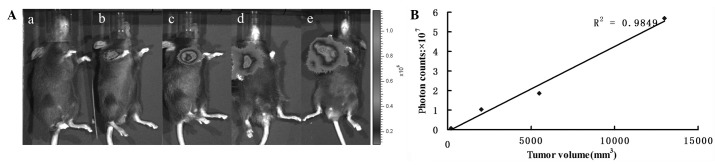
(A) Monitoring subcutaneously implanted RM-PSCA/Luc tumor cells using an *in vivo* bioluminescence imaging system. Visualized images were obtained extracorporeally (a) one, (b) two, (c) three, (d) four and (e) five weeks subsequent to the injection of 1×10^6^ RM-PSCA/Luc tumor cells. The respective photon counts of each mouse are represented by the scales beside the mouse images. (B) Correlation between bioluminescent signal intensity and tumor volume. Luc, luciferase; PSCA, prostate stem cell antigen.

All the mice inoculated with RM-PSCA/Luc cells exhibited a detectable tumor within two weeks, as assessed using bioluminescent reporter imaging. The bioluminescent emission registered at day 7 increased substantially from first appearance until day 34 (Fig. 3A). Quantification of the Luc signal was used for the *in vivo* monitoring of tumor growth. The correlation between the tumor size and photon counts was evaluated externally in the living mice. There was good correlation (R^2^=0.9849) between the photon counts and tumor volume (Fig. 3B); therefore, an *in vivo* imaging system may be used as a quantitative tool to monitor tumor growth. The bioluminescent signal was positively correlated with the tumor burden.

### Development of the tumor and the survival time of the mice

**Figure 4.**
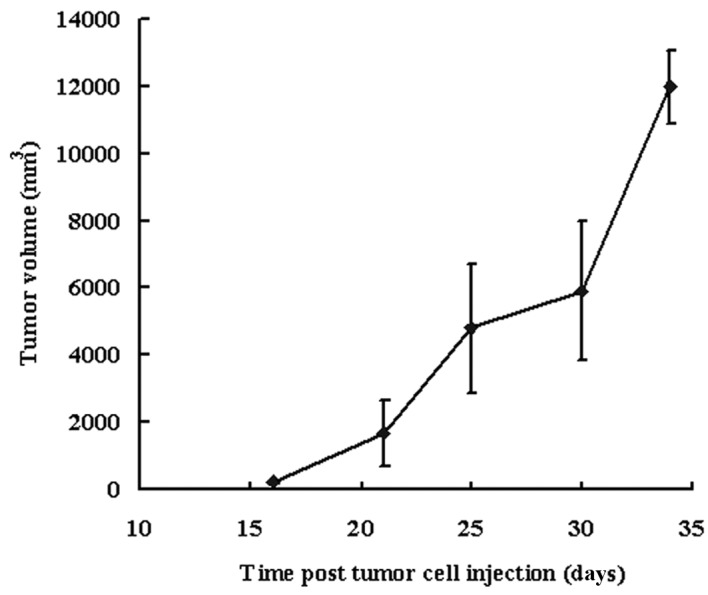
*In vivo* tumor growth of mice. RM-PSCA/Luc cells (1×10^6^) were injected subcutaneously into male C57BL/6 mice. The data are presented as the mean tumor volume (mm^3^) ± standard deviation. Luc, luciferase; PSCA, prostate stem cell antigen.

The tumor progressed quickly (Fig. 4) and the mean survival time of the mice was 38.4±3.05 days (34, 37, 39, 40 and 42 days).

### Immunohistochemical analysis

**Figure 5.**
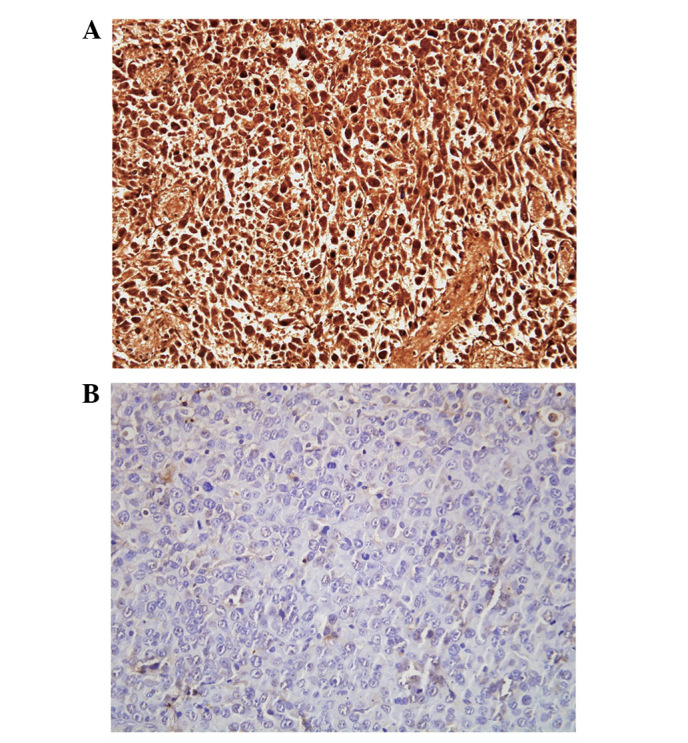
Detection of prostate stem cell antigen (PSCA) using immunohistochemical analysis. (A) Expression of PSCA in tumor tissues from the mice inoculated with RM-PSCA/Luc cells was positive with buff-colored staining (magnification, ×400). (B) Expression of PSCA in tumor tissues from the mice inoculated with RM-1 cells was negative with blue coloration (magnification, ×400). Luc, luciferase.

The cell line RM-PSCA/Luc was examined using immunohistochemical analysis. Tumor tissues with or without expression of PSCA were stained buffy (Fig. 5A) or blue (Fig. 5B), respectively. Analysis of the immunohistochemistry confirmed the presence of cancer cells in tumor tissues to be the sites of bioluminescent emission, detected using bioluminescent reporter imaging.

## Discussion

To the best of our knowledge, this study has, for the first time, presented a new model to enable the monitoring of prostate tumor growth using subcutaneous inoculation with Luc and PSCA-expressing RM-1 cells. The injection of RM-PSCA/Luc cells, combined with bioluminescent reporter imaging, may facilitate the early detection, continuous monitoring and quantitative localization of tumor growth *in vivo* in a noninvasive and sensitive manner.

To study the biological function of human PSCA and to evaluate the activities of anticancer drugs or vaccines for prostate cancer, we have established a traditional prostate tumor animal model with RM-PSCA cells and successfully evaluated the effect of a DNA vaccine based on PSCA and an HSP70 adjuvant (8,17). Traditional tumor animal models have a number of limitations, as follows: (i) Tumor growth and metastasis are not able to be visualized; (ii) there is a requirement for animals to be sacrificed at different time-points during the experiment, in order to obtain temporal information without consecutive study; (iii) the reflection of the time and space-expression of cells and genes is difficult. However, compared with the traditional tumor animal model, bioluminescent reporter imaging presents numerous advantages. It is time-saving and results in the generation of more data per experimental series, which leads to statistically sound results that are obtained more rapidly. Furthermore, it reduces the individual variation and the number of animals required (18). The orthotopic and allotopic tumor progression may be easily detected to monitor tumor growth and metastasis, without invasive procedures. Due to the predominance of Luc, bioluminescent reporter imaging for the detection of cells frequently reveals biological phenomena, including the progression process of definite gene expression, infectious diseases, tumor escape mechanisms and patterns of metastasis, and has been already generally applied in infection, gene therapy, organ transplantation, autoimmune disease, pharma projects, tumor immunity and treatment (19,20).

In this study, the RM-PSCA/Luc cell line with stable expression of PSCA and Luc was successfully constructed and was capable of establishing tumor growth *in vivo*. Compared with the athymic mouse, the C57BL/6 mouse has a normal immune system, which is favorable for evaluating the activities of anticancer drugs or vaccines.

In the present study, it was observed that RM-1 cells were transfected with PSCA and Luc genes without altering the efficacy of tumor establishment and growth. Following the subcutaneous inoculation of RM-PSCA/Luc cells, the subcutaneous tumor was able to be visualized using bioluminescent reporter imaging. However, tumor metastasis was not observed in this study. This may be due to the cell line or the fact that the tumor grew so quickly that the mice died prior to the occurrence of metastasis.

In conclusion, we have established a new model that utilizes the subcutaneous injection of Luc-reporter-positive transfected cancer cells. This RM-PSCA/Luc model, coupled with bioluminescent reporter imaging, provides a valuable experimental tool for the preclinical evaluation of the *in vivo* antitumor activity of investigational agents in the same animal. This model is likely to facilitate studies of the molecular mechanisms involved in the early stages of tumor progression and the development of new anticancer therapeutic strategies.
